# Using climate-FVS to project landscape-level forest carbon stores for 100 years from field and LiDAR measures of initial conditions

**DOI:** 10.1186/1750-0680-9-1

**Published:** 2014-02-04

**Authors:** Fabián B Gálvez, Andrew T Hudak, John C Byrne, Nicholas L Crookston, Robert F Keefe

**Affiliations:** 1 USDA Forest Service, Rocky Mountain Research Station, 1221 South Main St., Moscow, ID 83843, USA; 2 Department of Forest, Rangeland, and Fire Sciences, University of Idaho, 975 West 6th St., Moscow, ID 83844-1133, USA

**Keywords:** Carbon sequestration, Climate change, Forest vegetation simulator, General circulation model, Growth and yield, LiDAR

## Abstract

**Background:**

Forest resources supply a wide range of environmental services like mitigation of increasing levels of atmospheric carbon dioxide (CO2). As climate is changing, forest managers have added pressure to obtain forest resources by following stand management alternatives that are biologically sustainable and economically profitable. The goal of this study is to project the effect of typical forest management actions on forest C levels, given a changing climate, in the Moscow Mountain area of north-central Idaho, USA. Harvest and prescribed fire management treatments followed by plantings of one of four regionally important commercial tree species were simulated, using the climate-sensitive version of the Forest Vegetation Simulator, to estimate the biomass of four different planted species and their C sequestration response to three climate change scenarios.

**Results:**

Results show that anticipated climate change induces a substantial decrease in C sequestration potential regardless of which of the four tree species tested are planted. It was also found that *Pinus monticola* has the highest capacity to sequester C by 2110, followed by *Pinus ponderosa*, then *Pseudotsuga menziesii*, and lastly *Larix occidentalis*.

**Conclusions:**

Variability in the growth responses to climate change exhibited by the four planted species considered in this study points to the importance to forest managers of considering how well adapted seedlings may be to predicted climate change, before the seedlings are planted, and particularly if maximizing C sequestration is the management goal.

## Background

Forests cover about one third of the Earth’s terrestrial surface and have great capacity to store and cycle carbon (C). Living and dead wood, litter, detritus, and soil exceed the amount of C present in the atmosphere [1,2]. Forest resources supply a wide range of environmental services like mitigation of increasing levels of atmospheric carbon dioxide (CO_2_). Recent research shows how changes in forest cover and land use affect CO_2_ emissions to the atmosphere [3]. Evolution of new plant associations [4], shifts in the spatial distribution in tree species [5], redistribution of populations to local climates [6], and changes in site index [7] are the effects that climate change are having and are expected to have on forest ecosystems now and in the future. Tree growth, mortality and regeneration potential are typically adversely affected by the climate changing from the “normal” conditions to which tree species have adapted [8-10]. Conversely, climate change may lead to increased growth in other species [11] or other positive effects.

Ecosystem process-based models take the approach of simulating underlying biogeochemical processes, such as photosynthesis and respiration, using mathematical equations that determine the allocation of C from atmospheric CO_2_ into biomass. These models require parameterization for vegetation type, climate, and site conditions that constrain net primary productivity and ecosystem C balance. Forest-BGC (Biogeochemical Cycles) [12,13] and the Terrestrial Ecosystem Model (TEM) [14] partition C based on water and nitrogen limitations. The 3PG (Physiological Principles Predicting Growth) model has been linked to satellite image-derived estimates of canopy photosynthetic capacity to estimate forest growth [15,16]. Another alternative approach for assessing climate change impacts is to merge a state and transition model (STM) with the outputs from a dynamic global vegetation model such as MC1 [17] that predicts plant communities under equilibrium conditions. The MC1 model is so named because it combines biogeographic rules defined in the MAPSS [18] model with the CENTURY [19] biogeochemical model, which focuses on soil organic matter dynamics. The STANDCARB [20] model simulates both living and dead C pool dynamics at the forest stand level, which comes closer to what foresters expect as a measure of growth and yield. LANDIS-II (Forest Landscape Disturbance and Succession) [21,22] simulates landscape-level forest succession and disturbance processes, as well as forest management. All of the aforementioned models have the capacity to explore climate change effects on forest C sequestration, and most can operate in a spatially-explicit manner.

As opposed to the suite of process-based models favored by ecologists, forest managers traditionally use empirical models for predicting forest growth and yield. As climate is changing, forest managers have added pressure to obtain forest resources by following stand management alternatives that are biologically sustainable and economically profitable [23]. An empirical growth and yield model extensively used in the United States, the Forest Vegetation Simulator (FVS), is an approved quantification tool by the American Carbon Registry and is used broadly to predict forest stand dynamics. FVS operates at the individual tree level, simulating growth, mortality, and regeneration based on empirical studies. Forest managers use FVS to summarize and predict current and future forest stand conditions under different management alternatives, where outputs obtained from the model are used as inputs to forest planning models and other uses [24]. Other uses of FVS take into account how management and forest practices affect stand structure and composition, determine suitability for wildlife habitat, estimate hazard ratings for insect disease outbreaks or potential fires, and calculate consequent losses from these events. FVS is a powerful suite of models which has been linked to Forest Service forest inventory data bases and geographic information systems, evolving into a useful suite of tools for forest managers [25].

Climate-FVS is a recent improvement upon FVS that includes functions which take climate change and species-climate relationships into account when predicting tree growth, mortality, and regeneration establishment [8]. General Circulation Models (GCM) are specified within Climate-FVS since they are key to understanding future climates [26]. The variability in GCM outputs resulting from different model formulations and emissions scenarios are accounted for by running Climate-FVS such that different Climate-FVS runs are each informed by different GCM outputs. Neither FVS nor Climate-FVS currently have a spatial analysis capability.

Forest biomass and C stores and fluxes can be quantified at synoptic scales using remote sensing technologies, especially Light Detection And Ranging (LiDAR) [27]. Current commercial airborne LiDAR systems emit laser pulses of near-infrared light and measure the time elapsed until the light reflects off of the vegetation or ground and returns to the aircraft. Upwards of 100,000 laser pulses per second can be recorded, along with simultaneous inertial measurement unit (IMU) and global positioning system (GPS) measures of the aircraft position, to return a 3-dimensional point cloud characterizing at high resolution the x,y,z position of the ground and vegetation surfaces. LiDAR canopy height measures can be related to tree measures from forest inventory plots to map forest structure attributes of utility to forest managers [28]. Studies have applied LiDAR to extrapolate plot-level measures of forest biomass and C across forest landscapes [27,29], demonstrating the utility of area-based modeling methods for predicting (and mapping) current conditions.

While LiDAR and other remotely sensed data provide a snapshot of forest conditions in time, growth models such as FVS are commonly used to update the interval years between inventories, be they traditional field surveys or surveys that use both field and LiDAR or other remotely sensed data. Forest managers use FVS for planning purposes and for updating inventories under the assumption of an unchanging climate. This assumption of unchanging climate may be practical for predicting forest growth over the next 10 years or so. However, given the consensus among scientists that climate is changing, it is a difficult assumption to defend at longer time scales, such as a century, or the 50–80 year rotation length of managed, even-aged stands in the U.S. Northwest. In one previous study, Climate-FVS was employed to study the efficacy of active management alternatives applied in the aftermath of disturbances likely induced by climate change in the Rocky Mountains of Colorado, USA [30]. They concluded that adaptation-oriented management was necessary to provide for forest cover and accompanying C stocks during the 21^st^ century.

**Figure 1:**
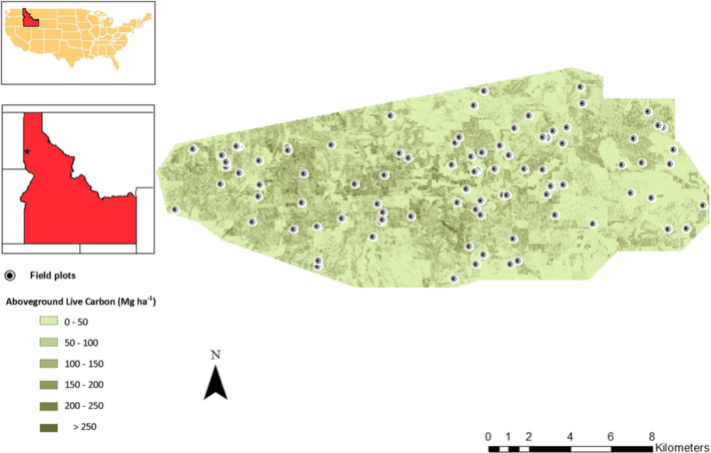
**Study area location and map of initial conditions in 2010 aboveground live C across the Moscow Mountain study landscape, derived following**[29]**from 2009 LiDAR and field plot data (locations overlaid).**

The primary objective of this study is to project the effect of typical forest management actions on forest C levels, given a changing climate, in the Moscow Mountain area of north-central Idaho, USA (Figure 1). The secondary objective is to upscale plot-level projections from a map of initial biomass conditions as mapped from 2009 LiDAR data across the 20,000 ha study area. Thus, results are summarized at two scales: At the plot level, the trees inventoried in 2009 are grown and summarized at decadal intervals for one century (2010–2110) using Climate-FVS. At the landscape level, the plot-level, decadal C projections are linked to a map of forest aboveground biomass predicted from the same forest inventory plot data and a 2009 LiDAR survey. Since forest management decisions are often made at the landscape level, the landscape-level projections may better inform a forest manager of the consequences of management alternatives on forest growth in the context of climate change. An implicit assumption in this study is that managers will want to sustain as productive a forest as possible by maximizing C sequestration on site.

## Methods

### Study area

Moscow Mountain is a western extension of the mixed-conifer forest type that dominates north-central Idaho, with agricultural lands more prevalent to the north, south, and especially Washington to the immediate west (Figure 1). Elevations range from 786 m to 1517 m, and annual rainfall from 630 to 1015 mm, with most precipitation falling during the winter and spring, while the summer and fall are dry. The common tree species, listed in order of decreasing drought tolerance [31] are: ponderosa pine [PP] (*Pinus ponderosa*), Douglas-fir [DF] (*Pseudotsuga menziesii*), western larch [WL] (*Larix occidentalis*), grand fir [GF] (*Abies grandis*), and western red cedar [RC] (*Thuja plicata*). Typical habitat types are the PP series at xeric sites on southern and western aspects, the DF and GF series on respectively moister sites, and the cedar/hemlock series on the most mesic sites on northern and eastern aspects [32]. A volcanic ash cap layer is thicker on northeast aspects and increases soil water holding capacity [33], augmenting the important influence of aspect on forest composition in this topographically complex landscape (Figure 1). Moscow Mountain is the setting of four large University of Idaho Experimental Forest management areas, extensive landholdings by private timber companies, as well as many private and some public land inholdings. The landscape is actively managed, with 26% of the 20,000 ha study area harvested between 2003 and 2009 alone [29].

### 2009 Forest inventory

#### LiDAR data

LiDAR data were collected 30 June 2009 at a mean density of 8.52 points/m^2^, including 50% overlap between adjacent flight lines limited to a scan angle of ±14° from nadir. A 4.3 cm vertical accuracy was achieved. Ground returns were classified using multiscale curvature classification [34], from which a 1 m resolution digital terrain model (DTM) was interpolated. LiDAR return elevations (Z) were normalized for topography by subtracting the DTM elevation from the LiDAR points, resulting in canopy height measures at every X, Y location sampled by the LiDAR. Canopy height, intensity, and density metrics characterizing the canopy structure were calculated at a cell resolution of 20 m across the entire LiDAR collection, along with topographic metrics from the DTM resampled to the same 20-m x 20-m (400 m^2^) cells [29]. The same suite of LiDAR metrics were also calculated from the normalized point cloud data and the DTM surface located within 89 fixed-radius field plots sampled across the study area.

#### Field data

Field plots for forest inventory measurements in 2008 (4 plots) or 2009 (85 plots) were distributed following a random stratified design based on topographic elevation, slope, aspect, and a Landsat satellite image-derived map of percent canopy cover [29]. The 89 plots were 400 m^2^ in size, within which all trees >10 cm were tallied. Saplings (≤10 cm and >1.37 m height) were tallied across the entire plot and seedlings (≤1.37 m height) within a 20 m^2^ subplot situated at plot center. Multiple plot center positions were logged using global positioning system (GPS) units with differential correction capability and averaged for an estimated plot location uncertainty of about one meter.

#### Forest aboveground biomass C map

Tree biomass estimates aggregated at the plot level were associated with the plot-level LiDAR metrics in an imputation model, with species-level plot biomass forming the response variables and the LiDAR metrics forming the explanatory variables. These plot-level field and LiDAR data comprised the reference plots for imputing forest aboveground biomass across the Moscow Mountain landscape, with the gridded LiDAR metrics representing the target cells where LiDAR data were available but field data were not. Imputation was used to assign the ground-measured attributes of the 89 plots to similarly sized target cells where no ground-measures were taken. The 89 plots are called reference observations and the gridded cells are called target observations [35]. In this case, the 400 m^2^ target cells were each assigned one of the 89 possible 400 m^2^ reference observations of total aboveground tree biomass. The act of making this assignment is an imputation. The assignments depended on the similarity of the LiDAR metrics in the target cells to those in the reference plots. The closest reference observation in a multivariate space defined by LiDAR metrics is its nearest neighbor. The exact definition of the multivariate space used in computing the distances is conditioned by the relationships between the LiDAR metrics and the biomass metrics that are evident in the 89 sample plots where both sets of metrics are known. An advantage of imputation is that it maintains the co-variance relationships between all plot attributes, meaning any measurements taken on a plot can also be imputed, even if they play no part in nearest neighbor selection. Therefore, the plot-ID corresponding to the imputed aboveground tree biomass reference observations mapped by [29] was itself mapped as an ancillary variable for this study.

### Climate-FVS

Like the standard FVS model, Climate-FVS reads initial stand or plot inventory information and uses it as starting values. In addition, Climate-FVS reads an additional input file that contains climate metrics (measures of temperature and precipitation as projected by downscaled GCM outputs) and species viability information that are specific to the location and elevation of the site being simulated. This additional file is generated using the “Get Climate-FVS Ready Data” webpage on the Rocky Mountain Research Station (RMRS) website [36] and requires a text file containing longitude, latitude, and elevation for each plot location. The mortality submodel increases mortality rates when tree species viability scores, ranging from 0–1, drop below 0.5 [8]. High-mortality rates may lead to the loss of some currently existing tree species in future years; Climate-FVS estimates that if viability scores decrease to <0.2, then the species is absent, with a chance of survival equal to zero. This study used Version 1 of Climate-FVS, which does not take into account genetically different populations of the same tree species for predicting mortality rates.

**Figure 2:**
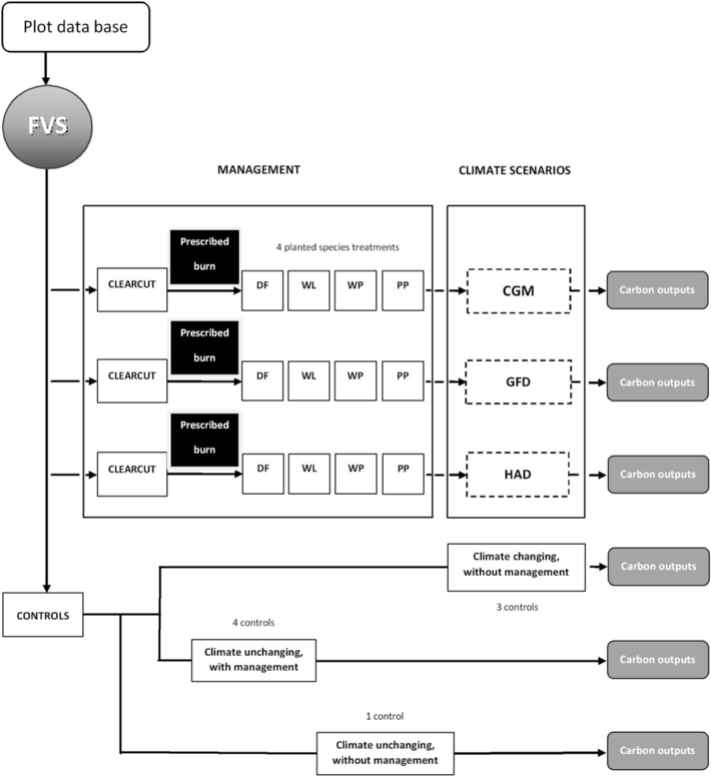
Schematic of management and climate scenarios projected for 100 years with Climate-FVS, including management and climate controls, based on 89 field plots, four tree species planted following management treatments, and three Global Circulation Models.

In this study, each plot was projected using a standard forest management option (clearcut harvesting initiated when stocking reaches 65% of normal, followed by prescribed burning and tree planting at a density of 200 trees/acre) under one of twelve treatments. The twelve treatments were combinations of four planted species (PP, DF, WL, WP (western white pine (*Pinus monticola*)) and three climate model outputs which form our climate change scenarios (Figure 2). The three GCMs used in this study are from the Canadian Center for Climate Modeling and Analysis Global Coupled Model (CGM), the Geophysical Fluid Dynamics (GFD) Laboratory at Princeton University, and the Met Office Hadley Centre (HAD) in the United Kingdom. Each climate change scenario corresponds to one GCM run according to the A2 emission scenarios [37] as described by [36]. We used the A2 emission scenarios assuming the highest levels rather than the lower B levels because current greenhouse gas levels are already higher than those contemplated when the climate model projections were made. The plot data were projected considering the four management treatments (plus one control) and three GCM scenarios (plus one control), or 5 x 4 = 20 projections per plot. In addition, a single control without management and without climate change was run for each plot. In this study, the Climate-FVS C projections for a given treatment and GCM combination varied solely as a result of the variability in initial conditions as measured across the 89 sample plots.

Tree growth from 2008–2009 tree diameter measures was projected to 2010 as a starting point, then projected for 100 years while summarizing at decadal intervals. Besides the standard FVS outputs (stem density, basal area, volume, etc.), two additional outputs were requested: The first is called the Carbon Report and provides the total aboveground live tree C as well as belowground live C, aboveground dead tree C, and total stand C. Also, it provides the C amount in the forest floor, forest shrubs, forest down dead wood, and the C that is removed by harvesting. The second special report is called the TREEBIO report and it provides biomass of live and dead trees, standing and removed trees, and breaks down the biomass into stem, crown, or live foliage. It returns estimated biomass in dry weight tons per acre, which is then multiplied by 0.5 to convert to C units [38].

### Computing area-wide totals

In a previous study [29], plot-level aboveground tree biomass at the 89 sample plots was measured and then those measurements were imputed to similarly sized map cells derived from the LiDAR, to form a study area-wide biomass map. Indeed, as stated above, those ground-based measurements are the same as the initial inventory data used here. A byproduct of the work by [29] was a data table that relates each of the 89 inventory plot projections to counts of the number of map cells in the study area to which each plot was imputed; these map cell counts served as weights in computing area-wide C projections. To map C projections across the study area (Figure 1), the plot-level C projections were joined to the map of imputed plot-IDs.

## Results

### Plot-level C sequestration

**Figure 3:**
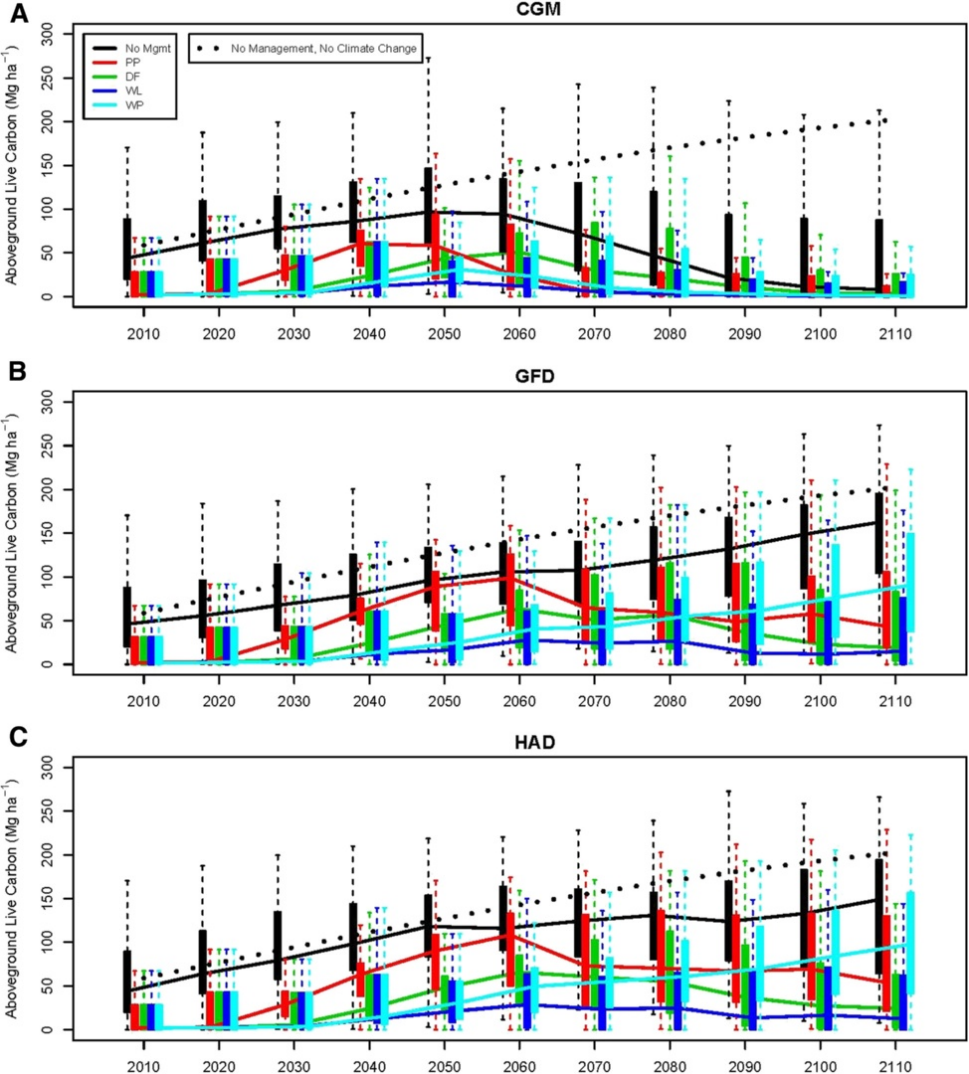
**Box and whisker plots of projected aboveground live C, with management treatments including plantings of four different tree species, under the A) Canadian Global Model (CGM), B) Geophysical Fluid Dynamics (GFD), and C) Hadley (HAD) climate change scenarios.** Lines connecting the boxplots indicate the medians, boxes indicate the 25^th^ to 75^th^ percentiles, and vertical dashed whiskers indicate the ranges calculated across the 89 plots at decadal intervals. The solid line control represents the climate scenarios without management. The dotted line control represents the mean trend of the 89 plots with no management and no climate change.

**Figure 4:**
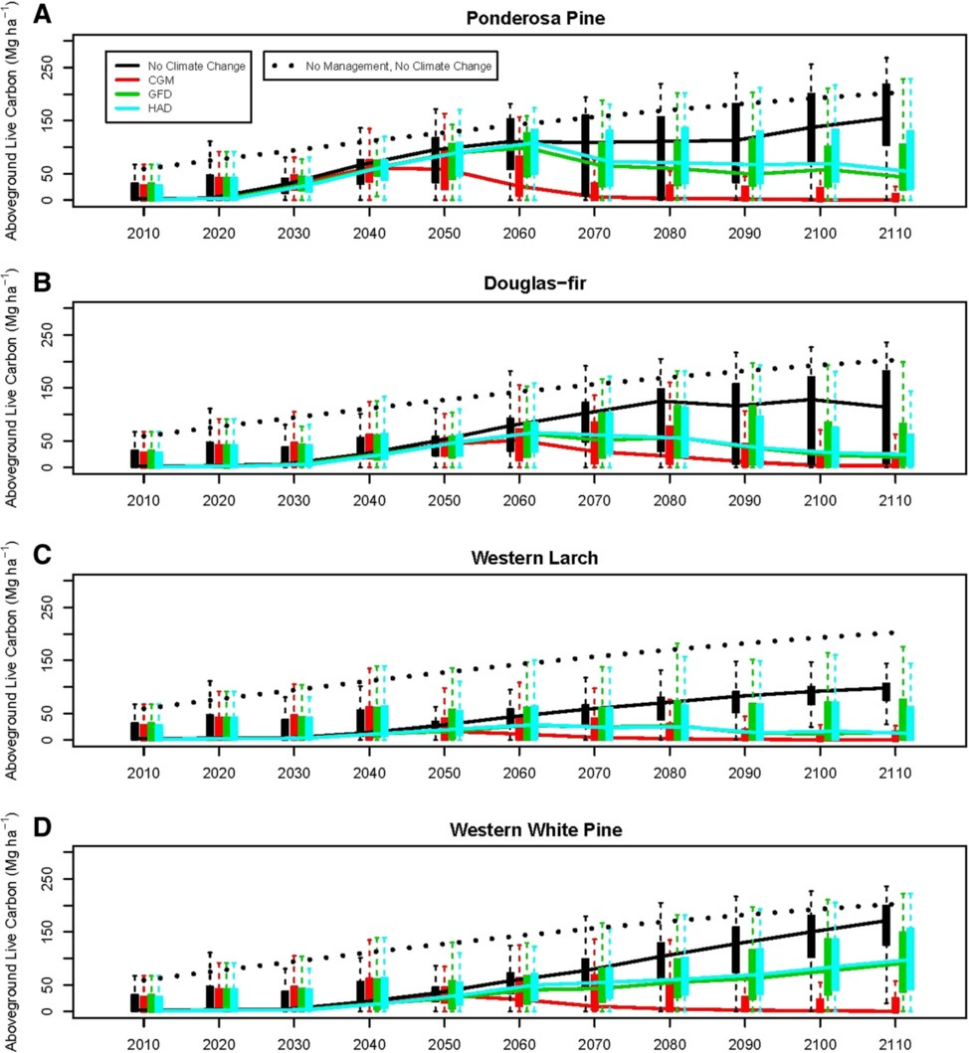
**Box and whisker plots of projected aboveground live C under four climate change scenarios with management treatments including plantings of A) ponderosa pine (PP), B) Douglas-fir (DF), C) western larch (WL), and D) western white pine (WP).** Lines connecting the boxplots indicate the medians, boxes indicate the 25^th^ to 75^th^ percentiles, and vertical dashed whiskers indicate the ranges calculated across the 89 plots at decadal intervals. The solid line control represents the planted species without climate change. The dotted line control represents the mean trend of the 89 plots with no management and no climate change.

Treatments and controls show a general tendency to increase C pools starting in 2010. Relative to the no management controls, management treatments always result in lower standing live tree C storage by 2110 regardless of tree species planted (Figures 3 and 4). Among these four species, PP plantings sequester C at the fastest rate in the first 50 years, but then C storage declines to 2110. DF and WL show the same trends but with less magnitude; DF peaks approximately a decade later, while WL trajectories are the least dynamic. Only WP steadily accumulates C to 2110, except under the CGM climate change scenario (Figures 3 and 4).

Similarly, C sequestration is invariably lower under all three climate change scenarios compared to the no climate change control. Among the three climate change scenarios, the CGM scenario results in the lowest C storage, while C levels are intermediate under the GFD and HAD scenarios (Figures 3 and 4). The mean of the full control projections that exclude both climate change and management is presented as a dotted line for reference in Figures 3 and 4.

**Figure 5:**
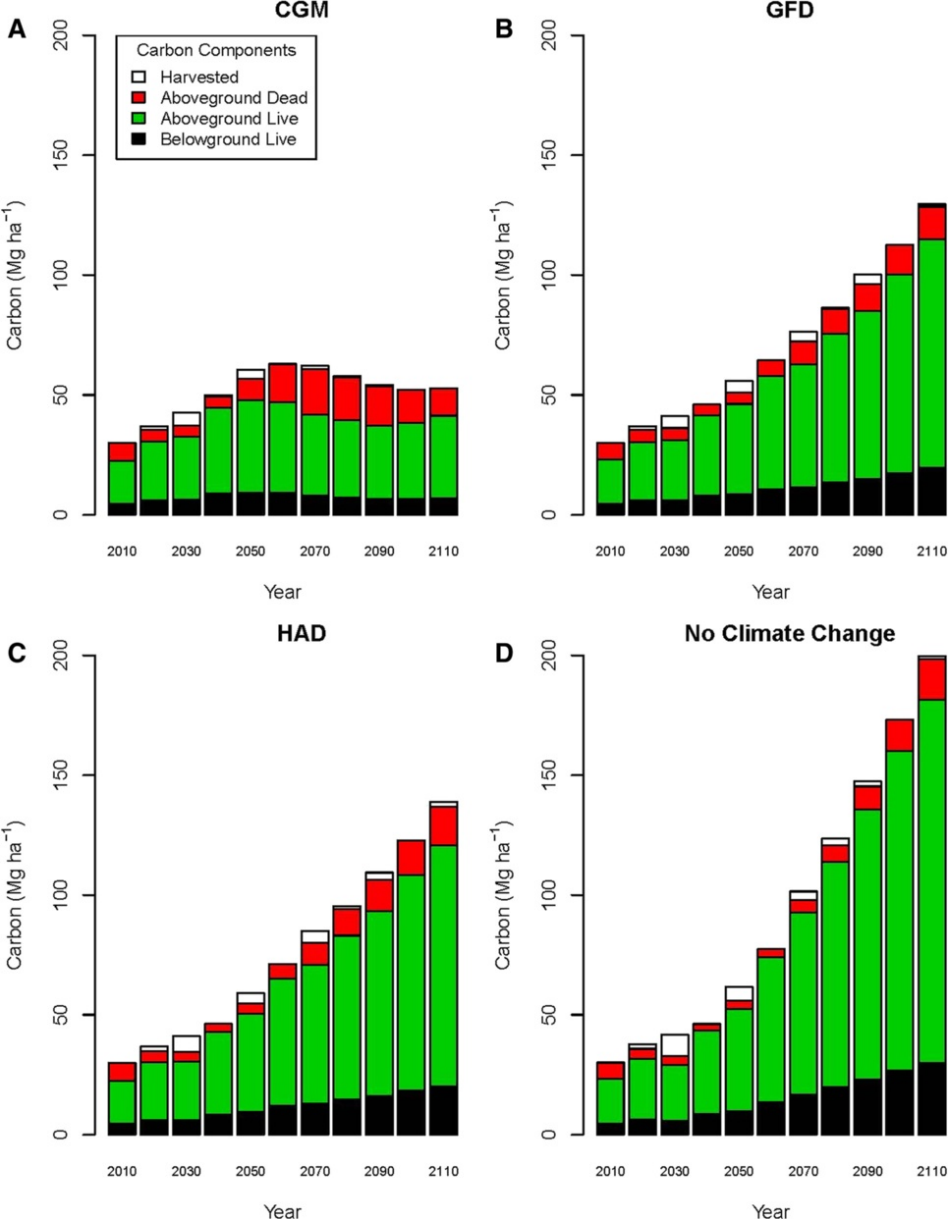
Plot-level means of C pools projected at decadal intervals with management treatments including plantings of WP under the A) Canadian Global Model (CGM), B) Geophysical Fluid Dynamics (GFD), C) Hadley (HAD), and D) No Climate Change scenarios.

Figures 3 and 4 illustrate plot-level projections for the aboveground live C pool, which is the largest of the C pools reported by Climate-FVS, but trends are similar for the other reported C components: belowground live, aboveground dead, and harvest removal (Figure 5). Among the four alternative plantings considered, planting WP sequesters the most C by 2110; it therefore comes closest to the control scenario that maximizes C sequestration, which is why we selected it as an illustrative example (Figure 5). Even if harvest removals are added to the other C pools that comprise total C on site, total C under every alternative climate scenario or management treatment is always less than the total C projected under the control scenario of no climate change. Because the C component pool trends remain consistent, the results reported in this paper focus on the aboveground live C, the most relevant C pool for managing forest C stores on site.

### Landscape-level C sequestration

**Figure 6:**
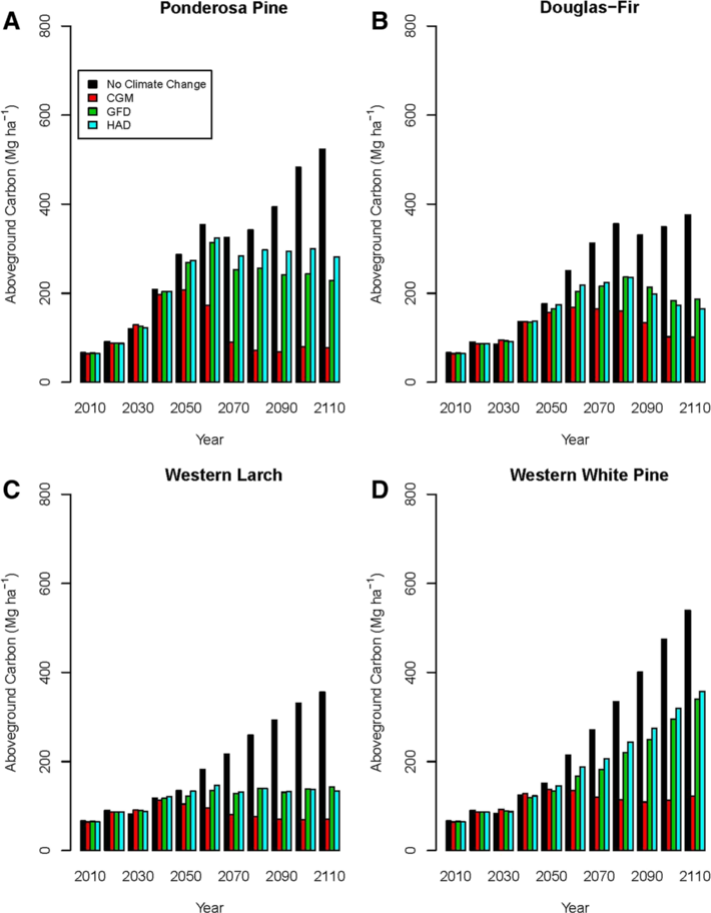
Column graph illustrating aboveground live C stores projected at decadal intervals under three climate change scenarios (plus control) and summed across the Moscow Mountain study landscape following management treatments with plantings of A) ponderosa pine (PP), B) Douglas-fir (DF), C) western larch (WL), and D) western white pine (WP).

Anticipated climate change induces a significant decrease in C sequestration potential regardless of which of the four tree species tested are planted (Figure 6). No climate change results in the greatest C storage regardless of tree species planted in the management treatments. C sequestration of PP plantings peaks earliest (2050–2060); DF plantings peak slightly later (2060–2080); WL plantings (besides the control) show the least amount of change; WP plantings produce the highest total C by 2110 of all four planted species in all three climate scenarios and the no climate change control; only the CGM scenario shows a decline, after peaking in 2050. Among the climate change scenarios (excepting the control scenario), HAD usually produces the largest C pools, followed closely by GFD, while CGM results in the lowest C storage.

**Figure 7:**
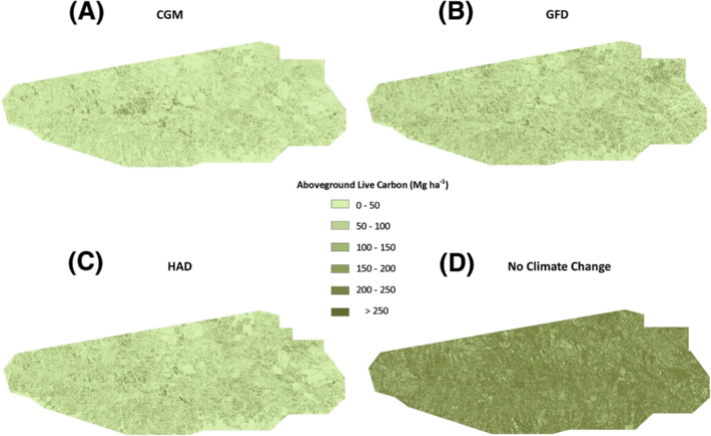
**Aboveground live C projected in 2110 across the Moscow Mountain study area, as exemplified by the management treatment that includes planting WP, per the A) Canadian Global Model (CGM), B) Geophysical Fluid Dynamics (GFD), C) Hadley (HAD), and D) No Climate Change scenarios.** A published map of aboveground tree biomass as predicted from field plots and a 2009 LiDAR collection (Figure 1) was used to define the same initial conditions used in all projections.

Figure 7 shows an example of the landscape-level maps summarized in Figure 6, with projected 2110 aboveground live C after management with WP plantings, under the scenarios of no climate change or the three GCMs. The spatial patterns in C as depicted in Figure 7 are invariant because no attempt was made to predict the location of management treatments. However, depending on which climate change scenario is considered, the overall magnitude of C sequestration is dramatically affected by 2110. Similar differences are evident if maps of the different tree plantings are compared, but are not included here for brevity, as the cumulative magnitude of landscape-level differences in C sequestration are already indicated in Figure 6.

## Discussion

For brevity, only the results for aboveground live C are presented in this paper, but the trends in the belowground live, aboveground dead, total, and other C pools reported in the FVS C report are similar, in that they are linked to the tree growth projections that comprise the growth engine that drives FVS and Climate-FVS [8,25]. Productivity rates drop dramatically when climate change is involved in the projections. Moreover, total production of wood volume at each simulated time step is the current standing volume of trees plus harvest removals for that timestep, which are ongoing over the simulation period. Tracking total production in this manner shows that a decrease in total production is due to the increased tree mortality projected by Climate-FVS, due to declining tree species viability scores. The Climate-FVS output shows that climate change is responsible for the detriment of the tree species initially present in the stands. The CGM climate scenario had the greatest impact among the three GCMs tested in this study.

Much of the following discussion considers elements of uncertainty. We highlight some specific issues emerging from this analysis that we feel are noteworthy, rather than several other potential sources of error – such as those related to the underlying FVS growth model as it is already broadly applied [24]. A few things are firmly understood and one of them is that climate is changing. While the GCM predictions differ, none predict that climate is not changing; furthermore, the magnitude of change is substantial. Another firm idea is that management decisions need to be made that depend on predictions about the future. Uncertainty, therefore, cannot be avoided. Climate-FVS is intended as a decision-making tool for forest managers despite the uncertainties implicit with climate change.

We caution that the uncertainties in these C projections are at least as high as the uncertainties in the GCMs themselves. This uncertainty would be expected to increase with time since the initial conditions were specified. Thus, it is more likely that PP may show the most favorable growth response to climate change in the next 50 years, than that WP may show the most favorable response in the next 100 years (Figures 3 and 4). More important is the trend that all four planted species show a depressed ability to sequester C if forecasted warming and drying occurs, than if it does not (control scenarios). Of the four planted species considered, WP shows the most resilience to long-term climate change effects on C sequestration, while WL shows the least resilience to climate change*.* This agrees with the particularly deleterious effects of climate change on WL as noted by [39], who predicted dramatic latitudinal and altitudinal shifts in the climate space suitable for WL. Forecasted rates of climate change are expected to exceed the rates that trees, especially WL, can migrate to their shifted climate space, bolstering calls for assisted migration [40] of seeds from distal sources. Seeds from different sources have different capacities to store C, but the expression of these differences depends on the environment [9,40,41]. Variability in the growth responses to climate change exhibited by the four planted species considered in this study points to the importance to forest managers of considering how well adapted seedlings may be to predicted climate change, before the seedlings are planted. These decisions may involve not just the tree species growing on Moscow Mountain, but also other species and spatially disjunct seed sources used to regenerate them.

There are additional sources of potential error in these simulations. As pointed out by [8], the Climate-FVS model uses empirically calibrated species-climate relationships based on the observed presence and absence of species. These data capture the realized niche of species that are due to competitive relationships between trees of different species as well as climate. The potential niche space is, by definition, larger than the observed, or realized, niche. Climate-FVS does not attempt to capture the potential niche effect and therefore it may overstate the effect of climate change on composition. On the other hand, there is a dearth of data that can be used to measure the largely unobservable potential niche.

In managed forests in the U.S. Northwest, investment in initial species establishment success following harvesting is required by law under state forest practices legislation. So, in general, managers have no choice but to conduct silvicultural treatments such as prescribed burning, planting, spraying, and subsequent replanting as needed, in order to successfully regenerate stands with target species composition. However, our results show that subsequent interactions among climate and productivity that affect intermediate stand development may still be evident. It is unclear how species-specific interactions of climate and stand productivity may manifest themselves in mixed stands where interspecific competition is also at play.

Other modeling approaches to addressing climate change arguably could be used instead of Climate-FVS. The principle shortcoming of FVS and Climate-FVS currently is that it does not ingest spatially explicit inputs or operate in a spatially explicit analysis framework, as do many ecosystem process-based models [e.g., [14,15,17,21,22]. Furthermore, forest-BGC [12,13], 3PG [15], and STANDCARB [20] were initially developed in and hence are well parameterized for U.S. Northwest forests. An application of MC1 in the U.S. Northwest [42] is particularly relevant in that it considers altered disturbance regimes due to climate change, and climate change effects on species assemblages. We view the fact that Climate-FVS operates at the level of individual tree species as an advantage for our study, because adaptation to climate change acts at the species level [10]. Landscapes like Moscow Mountain will likely remain a temperate coniferous forest for the next 100 years; it is the composition of that forest and rates of change in composition within that forest that are likely to change. Climate-FVS is arguably the best model to use in this context. We also chose to use Climate-FVS in deference to local managers’ desire to manage forests on Moscow Mountain for timber production. The four species we selected for planting following treatment are the most marketable tree species.

Our field plots sampled the Moscow Mountain landscape following a random stratified design, and simulated management treatments were distributed across the landscape according to their imputed plot-ID; this is why the plot-level and landscape-level projections in this study show similar trends and lead to the same conclusions. It would be more realistic and useful to allow the user to target specific locations (i.e., stands) for treatment, such as topographic positions with habitat types and tree species that may be more vulnerable to mortality from a warmer and/or drier climate. For instance, it is reasonable to assume that currently wetter NE aspects will remain wetter than SW aspects even as climate changes, because topographic variables are practically static even as climate is dynamic. Although we have linked forest growth projections to existing forest structure as characterized with LiDAR across the landscape, further research is needed to consider landscape context in future growth projections and management treatment alternatives. Indeed, the application of FVS and Climate-FVS within a spatially explicit modeling framework is an object of current development work.

## Conclusions

Different tree species sequester C at different rates, as constrained by genetics, site characteristics, and their interaction. Tree growth, total incremental production, and species viability are projected to be negatively affected by climate change in this mixed conifer forest in north-central Idaho, USA. While there are uncertainties in the GCMs, and differences between their outputs, there is little doubt that climate is changing. The projected declines in forest productivity and C sequestration potential under widely accepted climate scenarios deleteriously affect the four major commercial tree species most commonly planted in this environment. However, subsequent analyses to further understand potential climate impacts should distinguish among climate impacts on stand productivity, timing of individual stand harvests over the landscape, and optimally replanting individual stands to a variety of possible species or mixed-species alternatives over time in the context of landscape-level forest planning. Climate-FVS provides a powerful tool to forest managers regarding which trees to plant for mitigating the effects of global warming.

## Competing interests

The authors declare that they have no competing interests.

## Authors’ contributions

FG conducted analysis, summarized results, wrote initial draft. AH conceptualized and supervised the study, wrote most of the manuscript in its current form. JB trained FG to use Climate-FVS, graphed results, helped write. NC led development of Climate-FVS, selected climate change scenarios, helped write. RK selected management treatment scenarios, provided critical feedback, helped write. All authors read and approved the final manuscript.
